# Comprehensive Analysis of Clinically Relevant Copy Number Alterations (CNAs) Using a 523-Gene Next-Generation Sequencing Panel and NxClinical Software in Solid Tumors

**DOI:** 10.3390/genes15040396

**Published:** 2024-03-23

**Authors:** Vivek Gupta, Vishakha Vashisht, Ashutosh Vashisht, Ashis K. Mondal, Ahmet Alptekin, Harmanpreet Singh, Ravindra Kolhe

**Affiliations:** 1 Department of Pathology, Government Institute of Medical Sciences, Greater Noida 201310, India; dr_vivek_gupta@yahoo.com; 2 Department of Pathology, Medical College of Georgia, Augusta University, Augusta, GA 30912, USA; vvashisht@augusta.edu (V.V.); avashisht@augusta.edu (A.V.); amondal@augusta.edu (A.K.M.); aalptekin@augusta.edu (A.A.); hsingh1@augusta.edu (H.S.)

**Keywords:** Copy Number Alterations, NxClinical Software, Next-Generation Sequencing, Molecular Inversion Probe, FFPE Solid Tumor

## Abstract

Copy number alterations (CNAs) are significant in tumor initiation and progression. Identifying these aberrations is crucial for targeted therapies and personalized cancer diagnostics. Next-generation sequencing (NGS) methods present advantages in scalability and cost-effectiveness, surpassing limitations associated with reference assemblies and probe capacities in traditional laboratory approaches. This retrospective study evaluated CNAs in 50 FFPE tumor samples (breast cancer, ovarian carcinoma, pancreatic cancer, melanoma, and prostate carcinoma) using Illumina’s TruSight Oncology 500 (TSO500) and the Affymetrix Oncoscan Molecular Inversion Probe (OS-MIP) (ThermoFisher Scientific, Waltham, MA, USA). NGS analysis with the NxClinical 6.2 software demonstrated a high sensitivity and specificity (100%) for CNA detection, with a complete concordance rate as compared to the OS-MIP. All 54 known CNAs were identified by NGS, with gains being the most prevalent (63%). Notable CNAs were observed in *MYC* (18%), *TP53* (12%), *BRAF* (8%), *PIK3CA*, *EGFR*, and *FGFR1* (6%) genes. The diagnostic parameters exhibited high accuracy, including a positive predictive value, negative predictive value, and overall diagnostic accuracy. This study underscores NxClinical as a reliable software for identifying clinically relevant gene alterations using NGS TSO500, offering valuable insights for personalized cancer treatment strategies based on CNA analysis.

## 1. Introduction

Genomic instability, characterized by copy number alterations (CNAs), plays a pivotal role in cancer development and progression and in therapeutic resistance [1,2]. Amplifications and deletions within specific genomic segments can activate oncogenes or deactivate tumor suppressor genes, driving uncontrolled cell growth. Notably, solid tumors exhibit a heightened prevalence of CNAs, emphasizing their significance in the context of solid tumor biology [3]. While various laboratory-based methods, such as multiplex ligation-dependent probe amplification (MLPA) [4], microarray-based comparative genomic hybridization (aCGH) [5], SNP microarrays [6], RNA sequencing [7], fluorescence in situ hybridization (FISH) [8] and PCR-based approaches [9], facilitate CNA detection, they are often limited by factors like restricted coverage and resolution, tissue consumption, labor intensiveness, low throughput, and high cost [10]. Recent progress in next-generation sequencing (NGS) technologies has facilitated the concurrent identification of targeted CNAs and somatic mutations by utilizing a panel-based NGS approach. NGS technology is widely acknowledged for its proficiency in identifying single-nucleotide variants (SNVs) and small insertion and deletion variants (indels) [11]. Nevertheless, the accurate detection of CNAs remains a challenge when utilizing NGS data. This challenge arises from inherent limitations within the NGS technology, notably short read lengths and biases associated with GC content [3]. The complexities these factors introduce impede the reliable identification and characterization of CNVs, necessitating the exploration of alternative methodologies and analytical approaches to enhance the comprehensive assessment of genomic structural variations.

To address the existing constraints in the detection of CNAs, bioinformatics tools, such as CoNIFER, exomeCNV, CNVkit, and QDNA, have been developed, leveraging next-generation sequencing data [12,13]. While these tools demonstrate efficacy in reliably identifying large CNAs on the scale of megabases, their performance diminishes when confronted with smaller CNAs affecting only limited exonic regions [14]. Moreover, most of these tools are optimized for whole-genome or whole-exome datasets, presenting challenges when handling the sparser data generated by NGS gene panels routinely employed in genetic testing. The command line nature and reliance on a single executable file further complicate their integration into routine clinical settings [15,16]. Although benchmarks for CNA data-calling tools on targeted NGS panel data exist, these evaluations often utilize datasets from online repositories or predominantly rely on simulated data featuring a limited number of validated CNAs [17,18,19]. Hence, there is an imminent necessity to ascertain a tool capable of detecting clinical CNAs from NGS panel data with heightened precision and sensitivity.

Addressing this gap, the NxClinical platform (Bionano Genomics Inc., San Diego, CA, USA), that has been rebranded to VIA™ software Version 7.0, stands out as a software solution with an interactive graphical interface and compatibility with multiple file formats. This study explores the potential of an integrated workflow using NGS data and NxClinical 6.2 in detecting CNAs in formalin-fixed, paraffin-embedded (FFPE) solid tumor samples. Fifty (*n* = 50) previously well-characterized specimens by the OncoScan microarray inversion probe (OS-MIP) were used for this exhaustive validation and to assess the diagnostic precision of CNA detection through the proposed workflow.

## 2. Materials and Methods

### 2.1. Patient Samples

We analyzed a diverse set of 50 FFPE solid tumor cases, including breast cancer (*n* = 10), ovarian carcinoma (*n* = 8), pancreatic cancer (*n* = 8), melanoma (*n* = 4), and prostate carcinoma (*n* = 20). This study received approval from the Institutional Review Board (A-BIOMEDICAL I, IRB registration number 00000150, Augusta University; Human Assurance Committee IRB number 61129). Informed consent was obtained for newly collected samples, while waived authorization was granted for de-identified, banked samples. All protected health information was removed, and data were anonymized (coded and double-blinded) before study accession. OS-MIP confirmed the presence of pathogenic/likely pathogenic structural variants in all FFPE samples.

### 2.2. Tissue Selection and DNA Extraction

Hematoxylin and eosin (H&E)-stained FFPE tissue sections were reviewed by a board-certified pathologist to identify cancerous regions. Following this, macrodissection was performed on the tumor areas, enriching for higher tumor cellularity to achieve a minimum tumor percentage of 20%. DNA extraction used the QIAamp DNA FFPE tissue kit (Qiagen, Hilden, Germany) and DNA quality was checked by NanoDrop (ThermoFisher, Waltham, MA, USA). Double-stranded DNA was quantified using the Qubit DNA High Sensitivity (HS) assay kit (Life Technologies Waltham, MA, USA).

### 2.3. OncoScan Microarray Inversion Probe (OS-MIP)

For validation of CNA calling, all FFPE samples were tested in parallel by OS-MIP. Samples were characterized using the OncoScan™ FFPE Assay Kit (ThermoFisher, Waltham, MA, USA) with probes targeting specific genomic regions. Briefly, 80 ng DNA/sample was hybridized according to the manufacturer’s guidelines. Hybridized and processed samples were scanned through the GENECHIP Scanner-7G (Affymetrix, Santa Clara, CA, USA) to identify copy number and somatic mutation variations. 

### 2.4. Library Preparation for Next-Generation Sequencing 

The experimental procedure adhered strictly to the manufacturer’s guidelines for library preparation, employing the TruSight Oncology 500 Library Preparation Kit (Illumina, San Diego, CA, USA) based on hybrid capture principles [20]. The fragmentation of DNA was executed using an ultrasonicator (Covaris, Woburn, MA, USA), yielding DNA fragments ranging from 90 to 250 base pairs (bp) with a specific target peak at approximately 130 bp. Subsequent steps included end repair, A-tailing, and adapter ligation. The DNA fragments, now linked to adapters, were subjected to amplification through index PCR utilizing primers tailored to the UP-index. Further refinement was achieved through sample enrichment, employing probe-based hybridization (OPD2). The enriched samples underwent a series of procedures encompassing capture, PCR-driven enrichment, purification, and quantification of double-stranded DNA using the High Sensitivity Qubit kit (Q32854 Invitrogen, Waltham, MA, USA). Following bead-based library normalization, the normalized DNA libraries were combined to create a pooled sample, primed for ultimate loading onto the sequencer for downstream analysis. The TruSight Oncology 500 (TSO500) sequencing procedure encompassed a 101-base-pair paired-end sequencing methodology, employing 218 cycles. The library specimens were subjected to sequencing on Illumina’s NextSeq550 DX, utilizing the V2 flow cell kit (Illumina, San Diego, CA, USA).

### 2.5. Post-Sequencing Variant Analysis

The post-sequencing data analysis was performed using the BaseSpace TSO500 Assessment App (Illumina). Briefly, the output of the sequencing procedure produced FASTQ files, which were then converted into binary base call (BCL) files. These BCL files were subsequently processed through Qiagen’s QCI Cloud Connect software, converting them into variant calling format (VCF) files. These VCF files were then uploaded to the QCI Variant Interpreter platform, where advanced computational algorithms were applied to classify the variants. The classification process adhered to the collaborative guidelines set forth by the American College of Medical Genetics (ACMG) and the Association for Molecular Pathology (AMP), ensuring a standardized and rigorous assessment of the identified genetic variants.

### 2.6. CNA Analysis

**Figure 1:**
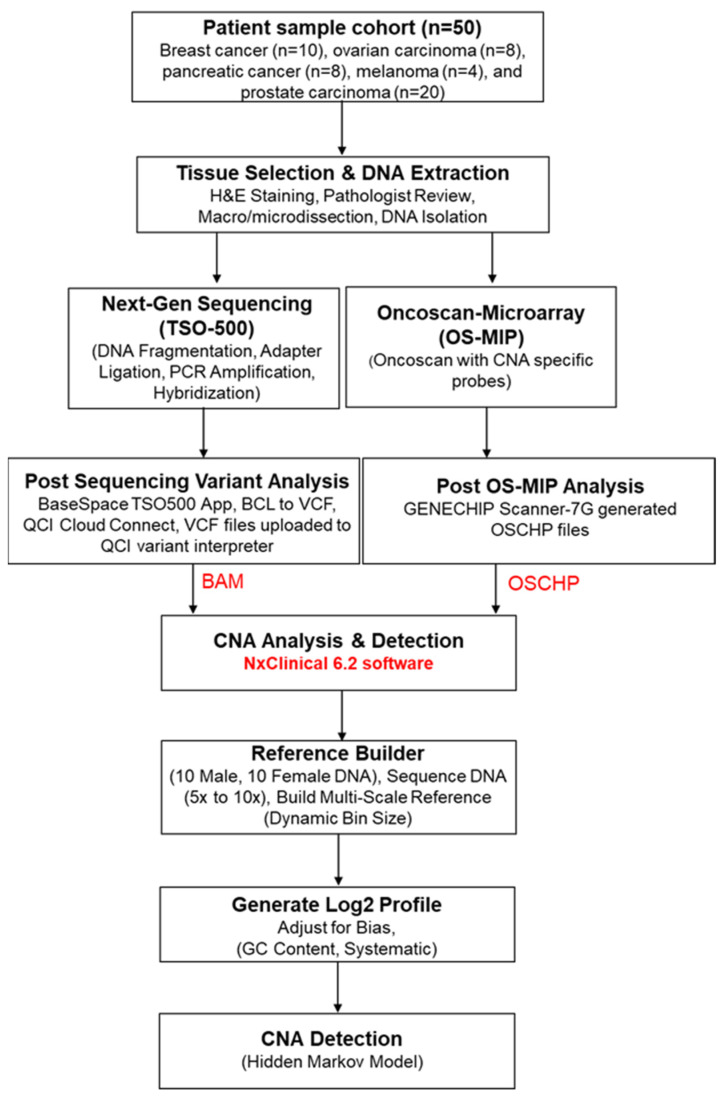
Workflow for copy number alteration (CNA) detection and analysis using Oncoscan Molecular Inversion Probe (OS-MIP) and Next Generation Sequencing (NGS) (TSO500) on NxClinical 6.2 software.

A total of 24 clinically relevant regions/genes were selected in this study for analysis after extensive literature review and expert consensus (Supplementary Table S1). CNAs were detected by analyzing binary alignment map (BAM) files obtained from Qiagen’s QCI Cloud Connect software and OSCHP files from OS-MIP using NxClinical 6.2 software (Bionano Genomics Inc., San Diego, CA, USA). To initiate the analysis, a reference profile was established utilizing genomic DNA extracted from a cohort of healthy controls (comprising 10 male and 10 female individuals). The DNA was sequenced at an approximate coverage range of 5× to 10× through the Binary Alignment Map Multi-Scale Reference Builder (Bionano Genomics Inc.) module. This algorithm adopts a dynamic bin size strategy to maintain a consistent number of reads per bin. In this context, a target of 1000 reads per bin was set. Subsequently, the reads per bin for each test sample were juxtaposed with the expected read count, and a series of systematic correction steps were applied. These adjustments accommodated various sources of bias, including GC content. The outcome of this process was the generation of a log2 profile of bins spanning the entire genome, centered on zero to denote no copy number change. CNAs were then detected using the hidden-Markov-model-based fast adaptive states segmentation (SNPFASST2) algorithm. This algorithm establishes hidden states for each CNA value based on anticipated log2 ratios and B-allele frequency, providing a comprehensive and accurate assessment of genomic copy number alterations. We did not include larger CNVs, particularly those exceeding 5 Mb, which may encompass vast stretches of non-coding or less biologically relevant regions of the genome. Gain was defined by one extra copy of a gene, so there are three copies instead of the normal two, and amplifications were defined by two or more extra copies of a gene, so four copies instead of the normal two. Loss is considered when one or both copies of genes were not present. The workflow for CNA detection is shown in Figure 1.

## 3. Results

### 3.1. Concordance of CNA Detection

**Figure 2:**
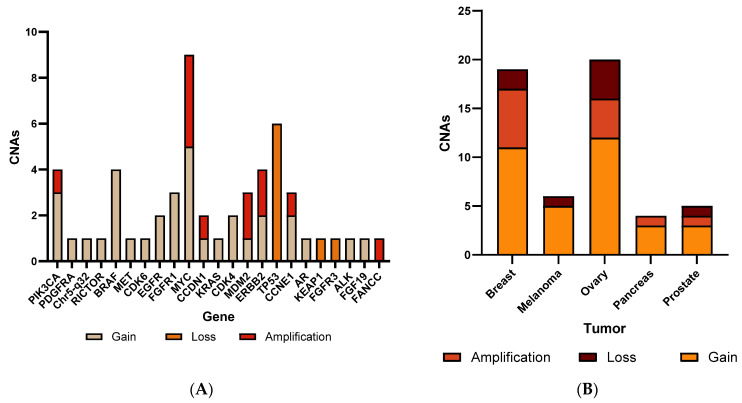
Bar chart of CNAs detected (**A**) in 24 cancer genes (**B**) in different tumor samples in 50 patients of the retrospective cohort, including breast, ovary, pancreas, melanoma, and prostate tumors.

A retrospective study was conducted to validate CNA identification using NGS on NxClinical software. We analyzed 50 FFPE samples, encompassing a diverse range of tumor types with varying tumor cellularity (20% to 100%). The male/female ratio was 1.2. Previously genotyped by the OncoScan, these samples were selected for the validation cohort, revealing complete concordance for CNAs between the NGS and OncoScan. In the examination of 24 regions for CNAs, out of the 54 known CNAs across the 50 samples, gain emerged as the most prevalent, accounting for 62.9% (34/54), while loss represented 14.8% (8/54) of total CNAs (Supplementary Table S2). Amplifications accounted for 22.2% (12/54) with NGS (Figure 2A). Figure 2B illustrates the distinctive CNA patterns observed across various tumors. Gains consistently emerge as the most prevalent CNAs across all tumor types. Notably, melanoma exhibits no instances of amplification, while pancreatic tumors show no loss.

### 3.2. CNA Patterns According to Tumor Type

**Figure 3:**
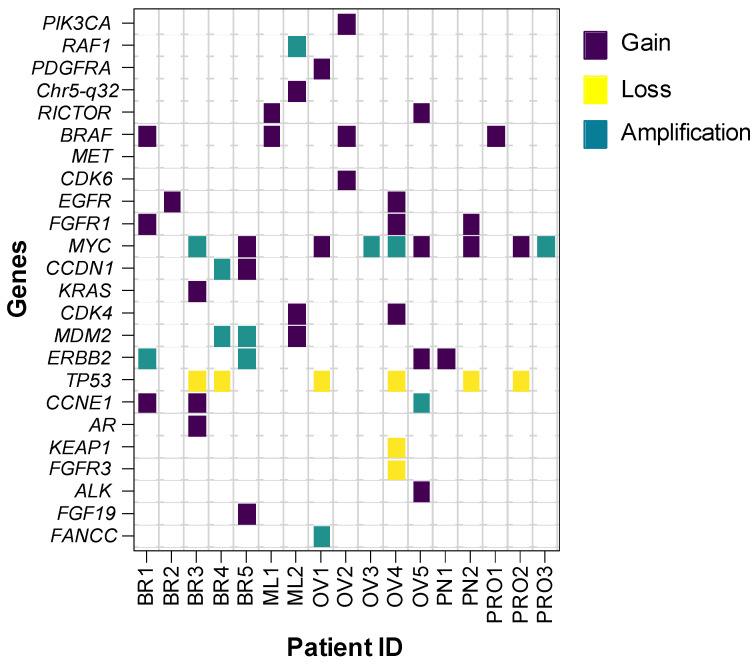
Heatmap of CNAs detected by NGS using NxClinical 6.2 software; BR = Breast; ML = Melanoma; OV = Ovary; PN = Pancreas; PRO = Prostate.

**Figure 4:**
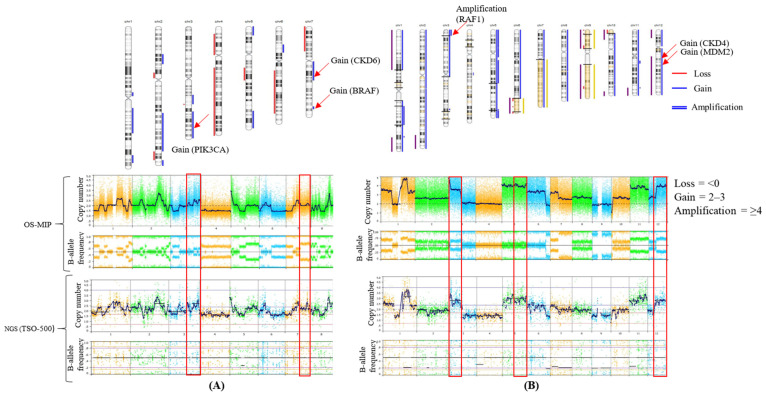
A representation of copy number analysis from NxClinical software version 6.2 using OS-MIP and NGS data; (**A**) shows 3q and 7q gain in ovary cancer sample (Case No. 14268) (**B**) shows 3q amplification 5q gain, 12q gain in melanoma sample (Case No. 14467). Gain/amplifications in specific genes are indicated by red arrows and boxes.

In breast cancer patients, CNAs were observed in 50% (5/10) of cases. The genes *MYC*, *CCDN*, *MDM2*, *ERBB2*, and *TP53* had the highest frequency of CNAs, each occurring in 20% (2/10) of patients. Meanwhile, *PIK3CA*, *BRAF*, *EGFR*, *FGFR*, *KRAS*, *AR*, and *CCNE1* were observed in 10% (1/10) of each. In 40% (4/10) of patients, two or more CNVs were observed simultaneously. CNAs were observed in two samples (50%, 2/4) in melanoma. *BRAF*, *RAF1*, *MDM2*, *RICTOR*, *CDK4*, and *MET* each occurred in 25% (1/4) of cases. For ovarian carcinoma tumors, CNAs were observed in five patients (62.5%, 5/8). MYC was prevalent in 50% (4/8) of cases, followed by *TP53* (25%, 2/8). *PDGFR*, *PIK3CA*, *BRAF*, *EGFR*, *FGFR1*, *RICTOR*, *FANCC*, *ALK*, and *CDK6* were each present in 12.5% (1/8) of cases. Four patients had two or more CNAs. In pancreatic tumors, CNAs were observed in 25% (2/8) of patients. *TP53*, *FGFR1*, *MYC*, and *ERBB2* each occurred in 12.5% (1/8) of cases. For prostate tumors, CNAs were observed in 15% (3/20) of patients. MYC had the highest frequency at 10% (2/20), followed by *TP53*, *BRAF*, and *PIK3CA*, each occurring in 5% (1/20) of cases. A heat map of CNAs detected by NGS using the NxClinical platform is shown in Figure 3. A representative copy number plot of the CNAs is given in Figure 4. 

### 3.3. Sensitivity, Specificity, and Accuracy

The sensitivity calculation for this method, derived from the analysis of 54 known CNAs in 50 samples, yielded a result of 100%, meaning no false negatives or false positives. Moving on to specificity, the assessment was based on 50 diagnostic routine samples, encompassing the analysis of 1200 individual genetic regions for CNAs within a target panel of 24 genes. Remarkably, no false positive results were detected, resulting in a specificity of 100%. As a result, the method exhibits an exceptional overall diagnostic accuracy, with both the positive predictive value (PPV) and negative predictive value (NPV) achieving a remarkable 100%, underscoring the method’s robustness, particularly in the context of negative predictions, as illustrated in Table 1.

## 4. Discussion

CNAs represent a significant and prevalent form of genomic diversity within the general population [21,22], with implications for various diseases, including autoimmune disorders, Alzheimer’s, and Parkinson’s [23,24]. Somatically acquired CNAs are particularly prominent in cancer, playing a substantial role in its pathogenesis [25,26,27]. The routine screening of FFPE samples from solid tumors for CNAs poses a considerable challenge, necessitating a testing platform compatible with low DNA quantity and high quality. As discussed above, traditional methods for assessing CNAs, including MLPA, SNP microarray, and techniques such as FISH, exhibit resolution and probe coverage limitations. The escalating demand for screening an increasing number of markers has prompted the integration of high-throughput technologies such as NGS and aCGH into clinical laboratories [28]. Also, the simultaneous detection of high-quality CNAs and sequence mutations from limited samples by these approaches streamlines the diagnostic process, providing a comprehensive and integrated assessment of genomic alterations and optimizes resource utilization. The precise detection of CNAs, especially in targeted NGS datasets, can enhance diagnostic yields and elevate the quality of clinical care.

While research has demonstrated the successful application of CNA detection algorithms to targeted NGS data, a lack of robust validation parameters has hindered their integration into diagnostic services. In this study, we proposed a streamlined workflow designed to facilitate the effective implementation of NxClinical software for CNA surveillance in targeted NGS datasets within a clinical setting. This approach aims to address the challenges associated with CNA detection in clinical environments, paving the way for improved diagnostic accuracy and patient care.

Recently, a complete concordance for detection of CNAs from 76 FFPE samples was observed between OS-MIP, NGS, and FISH [1]. In another study, a high degree of concordance (>97%) was observed between NGS with FISH [10]. However, fewer clinically relevant genes were included in the NGS panels of the above-mentioned studies. In a study by Chandramohan et al., 2022, the validation and implementation of tumor-only copy number alterations by a pipeline using CNVkit on a 124-gene panel for 28 pediatric solid tumors were performed. In this study, SCNA events involving a single exon (T19 RB1 exon 18) as well as events involving several exons (T26 ATRX exons 9 to 17) were studied. This highlights the potential for greater resolution of SCNA analysis when using sequencing data in comparison to clinical copy number arrays. The study was able to find SCNAs in 86% (24/28) of samples, with 46% (13/28) of samples harboring findings of potential clinical relevance [29]. In another study, researchers applied this integrated approach of NGS and an in-house algorithm for CNV detection in a retrospective cohort of 391 samples and a prospective cohort of 2375 samples and found a 100% sensitivity (95% CI, 89–100%) for 37 unique events. While in-house copy number variation (CNV) prediction algorithms boast complexity in their computational methodologies, their outcomes often lack consistency due to variations in assumed parameters and statistical reasoning. Consequently, their integration into clinical settings is hindered by this inconsistency, limiting their potential utility [30].

In the present study, 50 FFPE samples were analyzed on NxClinical software for detecting CNAs in numerous cancer-related genes using the 523 NGS gene panel from Illumina (TSO500). The CNA detection from NGS showed complete concordance with OS-MIP. NxClinical software is a user-friendly interface and accessible to users without any prior knowledge of bioinformatics or any command language. It also has the ability to analyze multiple file formats (OSCHP, CEL, BAM, VCF, etc.), enabling a centralized platform to consolidate analyses from multiple technologies. The CNAs tested included 24 clinically significant cancer-related genes in solid tumors of varying tissue of origin (e.g., breast, ovary, pancreas, melanoma, and prostate). Samples for this study were selected with a focus on 24 genes for which CNAs have well-established clinical implications, such as *MYC*, *MET*, *MDM2*, *TP53*, *ERBB2*, *EGFR*, *FGFR1*, etc. NGS exhibited 100% analytical sensitivity and specificity, precisely detecting 8 losses, 34 gains, and 12 amplifications in a set of 50 samples compared to the OS-MIP results. This underscores NxClinical software’s exceptional accuracy and reliability to detect CNAs from hybrid capture NGS library preparations. The genomic analysis of the present cohort revealed that CNAs were most frequently observed in *MYC* (18%), *TP53* (12%), *BRAF* (8%), *PIK3CA*, *EGFR*, and *FGFR1* (6%) genes. *MYC*, a proto-oncogene, governs crucial cellular functions in growth, proliferation, metabolism, differentiation, and apoptosis. Its amplification is frequently associated with malignancies, notably in breast cancer, where it emerges in advanced stages, signaling poor prognosis and increased risk of distant metastasis [31]. Our study identified a high frequency of CNAs in *MYC* in breast, ovary, and prostate cancer patients, shedding light on its potential role across multiple cancer types. *TP53* was suggested to be responsible for poor outcome and a higher number of genomic imbalances, corroborating the central role of chromosomal instability with respect to tumor aggressiveness and disease prognostication in younger breast cancer patients [32]. Notably, we observed *TP53* copy number loss is observed in ovarian, pancreatic, and prostate cancers, further linking it to tumorigenesis.

High frequencies of CNAs were observed in *PIK3CA*, *FGFR1*, and *EGFR* in breast, ovary, pancreas, and prostate cancer samples. Understanding these genetic alterations is crucial for personalized cancer treatment. Alterations in the *PI3K/AKT/mTOR* pathway, driven by changes in *PIK3CA*, promote cell proliferation and survival [33]. Elevated *FGFR1* expression or activity can contribute to uncontrolled cell growth and tumorigenesis. Understanding these genetic alterations is crucial for personalized cancer treatment. Targeted therapies designed to inhibit specific pathways, such as the *PI3K/AKT/mTOR* pathway or *FGFR1* signaling, may be considered for cancer patients exhibiting these genetic aberrations [34]. Also, *EGFR* copy number gain might be one of the accumulating genetic alterations during tumor progression and EGFR activation induced by *EGFR* copy number gain may contribute to tumor aggressiveness in triple-negative breast cancer [35]. In melanoma, the most frequent CNAs were observed in *BRAF* and previous studies suggested that an increased *BRAF* copy number could be associated with disease progression. Targeting *BRAF* mutations in melanoma has become a significant therapeutic approach. Drugs known as *BRAF* inhibitors, such as vemurafenib and dabrafenib, have been developed to specifically target and inhibit the activity of the mutated BRAF protein. These inhibitors can be combined with *MEK* inhibitors to improve their effectiveness [36].

Thus, the present study highlights the utility of a targeted NGS gene panel and the highly efficient NxClinical 6.2 software, which has been rebranded to VIA™ software in later versions, for comprehensive CNA analysis in routine clinical testing. This integrated workflow has the potential of providing insights into potential therapeutic targets and prognostic implications in various solid tumor types.

## 5. Strengths and Limitations

In the pursuit of advancing genomic analysis methodologies, this study delves into the application of NGS coupled with the NxClinical platform for the detection and interpretation of CNAs within predetermined genomic regions. While our investigation highlights several strengths and promising outcomes, it is imperative to acknowledge the inherent limitations that may impact the scope, generalizability, and robustness of our findings.

### 5.1. Strengths

1. Comprehensive Analysis: NGS platforms offer extensive genomic data, encompassing CNAs, SNVs, and other genetic aberrations. By leveraging NxClinical for CNA analysis within NGS data, the study consolidates multiple facets of genetic testing into a unified workflow. This integration facilitates a holistic understanding of the genomic landscape, enabling clinicians to gain insights into both structural and sequence-level alterations from a single analysis.
2. Streamlined Workflow: Integration of CNA analysis into NGS data processing through NxClinical enhances laboratory efficiency by streamlining workflow processes. This integration reduces the need for disparate tests, such as chromosomal microarrays, thereby optimizing sample processing time. The resultant expedited turnaround times contribute to timely generation of comprehensive genomic profiles, facilitating swift clinical decision making.
3. Cost Efficiency: Despite the inherent costs associated with NGS technology, the adoption of a unified NGS test for both CNA and sequence variant analysis can yield significant cost efficiency.

### 5.2. Limitations

1. Scope Limitation: The study’s focus on 24 predetermined genomic regions of clinical relevance within the NxClinical platform excludes other potential areas analyzed by the software. This selective approach may restrict the breadth of comprehensive CNA detection and interpretation. Clinicians interpreting study findings should acknowledge this scope limitation, which could impact the generalizability of the results to broader genomic landscapes.
2. Sample Size Inadequacy: With a sample size of 50, the study may lack the statistical power necessary to establish a coherent approach for a streamlined workflow utilizing NGS and NxClinical for CNA detection. A larger sample size is imperative to ensure robustness and generalizability of findings, particularly in understanding the nuances and complexities of genomic alterations.
3. Limited Cancer Type Validation: The validation of the NxClinical workflow was conducted with a restricted range of cancer types. Diverse categories of cancers should be included in future assessments to comprehensively evaluate the efficacy and accuracy of NxClinical in detecting CNAs across various malignancies.

## 6. Conclusions

In summary, our study demonstrated the efficacy of a targeted NGS gene panel in identifying CNAs within clinically relevant genes, including *MYC*, *MET*, *MDM2*, *TP53*, *ERBB2*, *EGFR*, and *FGFR1*, among others, utilizing NxClinical software with a remarkable specificity and accuracy rate of 100%. The findings exhibited comparable results to those obtained through OS-MIP. This underscores the importance of integrating CNA testing into routine NGS analyses, emphasizing its role in uncovering clinically significant gene alterations.

## Figures and Tables

**Table 1:** Diagnostic parameters calculations for copy number alteration (CNA) detection using NxClinical platform.

NxClinical (BAM Files TSO500)	NxClinical (OSCHP Files OncoScan)		
	POSITIVE	NEGATIVE	TOTAL
POSITIVE	54 (TP)	0 (FP)	54
NEGATIVE	0 (FN)	1146 (TN)	1146
TOTAL	54	1146	1200
Sensitivity TP/(TP + FN) = 100%	PPV TP/(TP + FP) = 100%		
Specificity TN/(FP + TN) = 100%	NPV TN/(FN + TN) = 100%		
Diagnostic accuracy (TP + TN)/TP + TN + FP + FN) = 100%			

TP = True Positive (positive outcome when the CNA is indeed present); TN = True Negative (negative outcome when the CNA is indeed absent); FP = False Positive (incorrectly indicates the presence of a CNA when it is actually absent); FN = False Negative (incorrectly indicates the absence of a CNA when it is actually present); PPV = Positive Predictive Value (likelihood that a positive CNA detection result accurately indicates the presence of that CNA); NPV = Negative Predictive Value (likelihood that a negative CNA detection result accurately indicates the absence of that CNA).

## Data Availability

All relevant data has been included in the manuscript.

## Supplementary Materials

The following supporting information can be downloaded at: https://www.mdpi.com/article/10.3390/genes15040396/s1, Table S1. List of 24 CNAs studied in the present study along with their potential cancer associated with them [32,34,35,36,37,38,39,40,41,42,43,44,45,46,47,48,49,50,51,52,53,54,55,56,57,58,59,60,61,62,63,64,65,66,67,68,69,70,71,72,73]. Supplementary Table S2. Categorization of CNAs detected by analysis in NxClinical with TSO500 and OncoScan.
