# Supplementary material for: Comprehensive Analysis of Clinically Relevant Copy Number Alterations (CNAs) Using a 523-Gene Next-Generation Sequencing Panel and NxClinical Software in Solid Tumors
Source: Genes (Basel). 2024 Mar 23;15(4):396. doi: 10.3390/genes15040396 (PMC11049607; doi:10.3390/genes15040396)
Supplement: Supplementary file 1 [file genes-15-00396-s001.zip › genes-2897169-supplementary.pdf]

genes

MDPI

Supplementary Material:

Supplementary Table S1. List of 24 CNAs studied in the present study along with their potential cancer associated with them.

S.No.	Studied genes/regions	Potential Cancer associations
1	ALK (2p23. 2–p23. 1)	Lung [37,38], Lymphoma [39]
2	PIK3CA (3q26)	Breast and Ovarian [40], Lung [41], Prostate [42]
3	RAF1 (3p26.3 - p25.2)	Ovarian [43], Lung [44]
4	PDGFRA (4q12)	Breast [45]
5	FGFR3 (4p16.3)	Ovarian [46]. Prostate [47]
6	Chr5q32	Breast [48,49]
7	RICTOR (5p13. 1)	Breast [50], Melanoma [51]
8	BRAF (7q34)	Melanoma [36], Ovarian and colorectal [52]
9	MET (7q21-q31)	Prostate [53], Melanoma [54]
10	CDK6 (7q21.2)	Melanoma [55], Ovarian [56], Prostate [65]
11	EGFR (7p11. 2)	Breast [35]
12	FGFR1 (8p11. 23)	Prostate [34]
13	MYC (8q24.21)	Pancreatic [57], Prostate [58]
14	CCDN1 (11q13)	Ovarian [59], Breast [60]
15	FGF19 (11q13)	Breast [61], Pancreatic [62]
16	KRAS (12p12.1)	Ovarian [63,64]
17	CDK4 (12q14.1)	Melanoma [55], Prostate [65]
18	MDM2 (12q14. 3-q15)	Pancreatic [66], Ovarian [67]
19	FANCC (16q24.3)	Breast [68,69]
20	ERBB2 (17q12)	Breast [70]
21	TP53 (17p13)	Breast [32]
22	CCNE1 (19q12)	Melanoma [71]
23	KEAP1 (19p13.2)	Melanoma [72], Prostate [73]
24	AR	Prostate [73]

Supplementary Table S2. Categorization of CNAs detected by analysis in NxClinical with TSO500 and OncoScan

Workflow	Gain	Amplification (High copy gain)	Loss	Total CNAs
NxClinical-TSO500	34	12	8	54
NxClinical-Oncoscan	34	12	8	54
